# Health related quality of life of dialysis patients in Malaysia: Haemodialysis versus continuous ambulatory peritoneal dialysis

**DOI:** 10.1186/s12882-019-1326-x

**Published:** 2019-04-30

**Authors:** Naren Kumar Surendra, Mohd Rizal Abdul Manaf, Lai Seong Hooi, Sunita Bavanandan, Fariz Safhan Mohamad Nor, Shahnaz Shah Firdaus Khan, Loke Meng Ong, Abdul Halim Abdul Gafor

**Affiliations:** 1 Department of Community Health, Faculty of Medicine, Pusat Perubatan Universiti Kebangsaan Malaysia, Jalan Yaacob Latif, Bandar Tun Razak, 56000 Kuala Lumpur, Cheras Malaysia; 2 Hospital Sultanah Aminah, Jalan Persiaran Abu Bakar Sultan, 80100 Johor Bahru, Johor Malaysia; 3 Hospital Kuala Lumpur, 50586 Jalan Pahang, Kuala Lumpur, Malaysia; 4 Hospital Tengku Ampuan Afzan, 25100 Kuantan, Pahang Malaysia; 5 Hospital Tengku Ampuan Rahimah, Jalan Langat, 41200 Klang, Kuala Lumpur, Selangor Malaysia; 6 Hospital Pulau Pinang, Residency Road, 10990 Penang, Malaysia; 7 Nephrology Unit, Faculty of Medicine, Pusat Perubatan Universiti Kebangsaan Malaysia, Jalan Yaacob Latif, Bandar Tun Razak, Cheras, 56000 Kuala Lumpur, Malaysia

**Keywords:** Dialysis, Continuous ambulatory peritoneal dialysis, EQ-5D, Haemodialysis, Malaysia, Quality adjusted life year, Quality of life

## Abstract

**Background:**

Health related quality of life (HRQOL) is an important predictor of clinical outcomes for End Stage Renal Disease (ESRD) patients and to establish quality adjusted life years (QALYs) for economic evaluation studies. This study aims to measure the health utilities and to identify socio-demographic and clinical factors associated with HRQOL for haemodialysis (HD) and continuous ambulatory peritoneal dialysis (CAPD) in Malaysia.

**Methods:**

A total of 141 patients (77 HD and 64 CAPD) from 1 federal and four state hospitals participated in this cross-sectional study. Patients were randomly selected from the National Renal Registry (NRR) using a stratified random sampling. The EQ-5D-3 L questionnaire was used to measure HRQOL. Variables investigated include dialysis modalities, sociodemographic characteristics, co-morbidities and biochemical markers. Utilities are measured on an ordinal scale of 0–1, where 1 indicates full health and 0 indicates death.

**Results:**

The mean utility scores were 0.854 ± 0.181 and 0.905 ± 0.124 (*p* > 0.05) and the mean Visual Analogue Scale (VAS) scores were 76.2 ± 12.90 and 77.1 ± 10.26 (*p* > 0.05) for HD and CAPD patients respectively. There was a significant difference in problems reported between HD (35.1%) and CAPD (15.6%) on usual activities dimension (*p* = 0.009). The proportion of patients having problems in the pain/discomfort domain in both modalities was high (34.0%). Haemoglobin (< 10 g/dL) (*p* = 0.003), number of co-morbidities ≥3 (*p* = 0.004) and wheelchair-bound status (*p* < 0.001) were significant predictors of poor HRQOL.

**Conclusions:**

The present cross-sectional study shows that CAPD patients have a higher utility index score than HD patients but this was not statistically significant. The utilities index score may be used to calculate QALYs.

## Background

Health is defined by the World Health Organisation (WHO) as a state of complete physical, mental, and social well-being, and not merely the absence of disease or infirmity [1]. Since chronic diseases have an impact on health-related quality of life (HRQOL), this has become a key outcome measure in disease management [2, 3]. HRQOL is a multi-dimensional model that includes domains related to physical, mental, emotional and social functioning.

Patients with end stage renal disease (ESRD) require renal replacement therapy (RRT) in the form of dialysis or a kidney transplant. Kidney transplantation may offer a nearly normal life and is considered the optimum treatment for eligible patients [4]. Despite kidney transplants from live and deceased donors, organ shortage remains a worldwide problem producing increasing waiting lists for transplantation and necessity for dialysis treatments [5]. Alternative dialysis modalities are haemodialysis (HD) and peritoneal dialysis (PD). In Malaysia, kidney transplantation is very limited and the majority of ESRD patients require dialysis, either HD or PD, to sustain life [6].

There were 37,183 patients dialysing in 2015 (1220 per million population) [7]. Dialysis is financed through a mixed public-private model. Almost all PD patients (97%) were being treated in public settings [7]. There is a mixed financing system in dialysis provision in Malaysia with public financing is funded through the general taxation. The government also provide financial assistance to eligible patients through a few other agencies including SOCSO, a social welfare insurance body. Within the private sector, individuals can purchase health insurance on voluntary basis [6].

There are many complications associated with ESRD including anaemia, mineral bone disorders, cardiovascular disease and malnutrition [8]. As a result, ESRD has received increased attention as a leading public health problem. Estimates of the global burden of disease reported that kidney disease accounted for 1,129,000 deaths and 38,104,000 disability-adjusted life years (DALYs), making it the 12th highest cause of death (2.0% of all deaths) and the 19th cause of disability (1.4% of all DALY) [9].

ESRD patients on dialysis have impaired HRQOL which affects different domains of the patients’ lives [10–17]. A wide range of general and disease-specific questionnaires have been utilised for evaluating the HRQOL of patients with ESRD [10, 18]. In Malaysia, a limited number of studies were conducted to examine the HRQOL of dialysis patients using different questionnaires [15, 16, 19]. However, these studies were conducted mainly for patients’ clinical management. HRQOL assessment is not only important for comparing outcomes of healthcare interventions but for supporting decisions regarding the allocation of resources [20]. EQ-5D, a generic instrument for measuring HRQOL, is commonly used for health economics studies [21]. EQ-5D output includes health profiles derived from the descriptive system, self-rated health status derived from the Visual Analogue Scale (VAS), and a weighted index derived using preference weights to produce quality-adjusted life-years (QALYs) [22]. QALY is recommended by the National Institute for Health and Clinical Excellence (NICE) as a measure of health benefit for their ‘reference case’ and to enable a standardised approach for comparing economic evaluations across different healthcare areas [23].

Hence, the aims of the study are to estimate health utilities, to compare the HRQOL of HD and CAPD patients, and to identify factors associated with HRQOL through an analysis of socio-demographic and clinical variables using EQ-5D-3 L questionnaire.

## Methods

### Sample

This was a multi-centre cross sectional study conducted as part of the Cost Utility Analysis of End Stage Renal Disease Treatment in Ministry of Health (MOH) Dialysis centres, Malaysia: HD versus CAPD. The sampling frame for the selection of participating centres was MOH state hospitals that submit dialysis patients’ data to the National Renal Registry (NRR). A principal site investigator, sub-investigators and research assistants were appointed at each centre. Patients were eligible if they were above 18 years old, commenced dialysis between 2011 and 2015 and dialysis treatment is subsidised by MOH. Patients were excluded if they died, underwent kidney transplant, switched dialysis modality or transferred to a new centre during the study period. Patients were sampled by a stratified random sampling using the list of prevalent dialysis patients obtained from NRR. All patients provided written informed consent for participation in this study. One hundred and seventy three dialysis patients (90 HD and 83 CAPD) were recruited from 1 federal and 4 large state hospitals. The study period for cost utility analysis was from 1st October 2016 to 30th September 2017. HRQOL data was collected during the last quarter of the study period (May 2017 to September 2017).

### Recruitment and data collection procedures

Consent was taken by the principal investigator or the principal site investigator at each site. Upon signing the consent form, socio-demographic details were collected including education level, monthly household income, body mass index (BMI) and employment status. Duration on dialysis, and co-morbidities were assessed from patients’ medical records.. Patients were described of having hypertension and/or diabetes through self-reporting diagnosed by medical personnel or on respective medication. For *Systemic lupus erythematosus* (SLE), it was identified according to the American College of Rheumatology (ACR) 1997 guidelines. A patient who fulfilled least four of the eleven criteria was accepted as having lupus [24]. Patients were classified as having cardiovascular disease (CVD) when they had history of coronary artery or cerebrovascular disease as diagnosed by medical personnel. A *positive HBsAg test* result indicates that the patient is infected *with Hepatitis B* and a *positive* anti-HCV result indicates that the patient is infected *with Hepatitis C.* Laboratory test results were also recorded including serum calcium (mmol/L), haemoglobin (g/dL), serum albumin (g/L) and dialysis adequacy (Kt/V). Patients’ HRQOL was measured using the EQ-5D-3 L questionnaire developed by the EuroQol group. This questionnaire is validated for Malaysia’s healthcare setting [22]. The EQ-5D questionnaire comprises a visual analog scale (VAS) and an EQ-5D descriptive system. Respondent’s self-appraised health status is captured using VAS which is *graduated* in 10-points increments from *0* (worst imaginable health state) to *100 (best imaginable health state)*. The descriptive system contains 5 health dimensions; mobility, self-care, usual activities, pain/discomfort, and anxiety/depression. It can be used as a health profile or transformed into an index score. The questionnaire is available in four languages including English, Malay, Chinese and Tamil. Since the questionnaire is self-administered, patients were asked to complete the form during a regular HD session (for HD) or scheduled clinic visit (for CAPD). When patients had trouble reading, writing or understanding the questionnaires, a trained research assistant provided assistance.

### Statistical analysis

Continuous variables were expressed as mean ± standard deviation (SD). Discrete variables were reported as frequency and percentage. Chi-square test or Fisher’s Exact test were used for group comparisons (HD and CAPD) where appropriate. For EQ-5D-3 L utility index and VAS, the distributions of scores were reported as the mean ± (SD) and median, minimum, maximum, and the percentage of problems reported. Malaysian tariff developed by Faridah Aryani et al. was used to convert the utility score (N3 Rescaled VAS scoring algorithm) [23].

In the bivariate analysis, continuous independent variables were converted into categorical variables since there was a non-linear relationship between the dependent variable (utility index score and VAS score) and the continuous independent variables. Continuous variables were coded using either recommended clinically meaningful values or means/medians when the former was absent. Since the data of utility scores and VAS score were not normally distributed, on the basis of the Kolmogorov-Smirnov and the Shapiro-Wilk tests of normality (*P* < 0.001), non-parametric statistical tests were performed. In the multivariate analysis, the one-way analysis of covariance (*ANCOVA*) was used. Only statistically significant factors in the bivariate analysis were entered into the model. The model fit was tested according to the following assumptions; a) random sample, b) independence of observations c) approximate normality and d) equal variance. Assumptions a and b were related to study design which were fulfilled. Normality and equal variance (*homoscedasticity)* assumptions were tested using scatter plots and Levene’s test of the standardised residuals. A *p* value < 0.05 was considered significant. All tests were conducted using SPSS version 24.

### Ethics approval

Ethical approvals were sought from the Institutional review board of UKMMC (Fundamental FF-2016-288) and Medical Research Ethics committee (MREC) (NMRR-16-1341-30,856). This study was also registered at ClinicalTrials.gov (NC T02862717).

## Results

One hundred and forty-one patients answered the EQ-5D-3 L questionnaire. The study patient characteristics are shown in Table 1.

**Table 1:** Patient characteristics

Characteristics	All patients (*n* = 141)	HD (*n* = 77)	CAPD (*n* = 64)	*P* value
Age (years), mean (SD)	53.7 (14.20)	53.9 (14.90)	53.5 (13.43)	0.830^a^
Age group, n (%)				0.100^b^
Young, 18–45	36 (25.5)	19 (24.7)	17 (26.6)	
Middle aged, 46–65	75 (53.2)	41 (53.2)	34 (53.1)	
Elderly, > 65	30 (21.3)	17 (22.1)	13 (20.3)	
Gender, n (%)				0.066^b^
Male	78 (55.3)	48 (62.3)	30 (46.9)	
Female	63 (44.7)	29 (37.7)	34 (53.1)	
Ethnicity, n (%)				0.335^b^
Malay	65 (46.1)	32 (41.6)	33 (51.6)	
Chinese	51 (36.2)	32 (41.6)	19 (29.7)	
Indian/others	25 (17.7)	13 (16.9)	12 (18.8)	
Household income per month (RM), n (%)				0.453^b^
< 3000	114 (80.9)	64 (83.1)	50 (78.1)	
≥ 3000	27 (19.1)	13 (16.9)	14 (21.9)	
Education level, n (%)				0.100^b^
Primary	39 (27.7)	19 (24.7)	20 (31.3)	
Secondary	84 (59.6)	44 (57.1)	40 (62.5)	
Tertiary	18 (12.8)	14 (18.2)	4 (6.3)	
Occupation, n (%)				0.634^b^
Employed	38 (27.0)	22 (28.6)	16 (25.0)	
Unemployed/Retired/Housewife	103 (73.0)	55 (71.4)	48 (75.0)	
Primary Renal disease, n (%)				0.966^b^
Diabetes mellitus	65 (46.1)	34 (44.2)	31 (48.4)	
Hypertension	32 (22.7)	17 (22.1)	15 (23.4)	
SLE */* Glomerulonephritis	24 (17.0)	14 (18.2)	10 (15.6)	
Polycystic kidney	7 (5.0)	4 (5.2)	3 (4.7)	
Unknown cause /Others	13 (9.2)	8 (10.4)	5 (7.8)	
Dialysis duration (years), mean (SD)	3.9 (1.43)	4.1 (1.46)	3.7 (1.37)	0.114^a^
Co-morbidities, *n* (%)				
Diabetes mellitus	69 (48.9)	36 (46.8)	33 (51.6)	0.570^b^
Hypertension	82 (58.2)	46 (59.7)	36 (56.3)	0.654^b^
SLE	6 (5.0)	3 (3.9)	3 (4.7)	1.000^c^
CVD	20 (14.2)	13 (16.9)	7 (10.9)	0.314^b^
Hepatitis B infection	10 (7.1)	10 (13.0)	0 (0)	**< 0.001** ^b^
Hepatitis C infection	6 (4.3)	6 (7.8)	0 (0)	**0.032** ^c^
Body Mass Index (kg/m^2^), *n* (%)				0.134^b^
Underweight (< 18.5)	16 (11.3)	13 (16.9)	3 (4.7)	
Normal weight (18.5–24.9)	73 (51.8)	37 (48.1)	36 (56.3)	
Overweight (25.0–29.9)	31 (22.0)	15 (19.5)	16 (25.0)	
Obese (≥30)	21 (14.9)	12 (15.6)	9 (14.1)	
Biochemistry, mean (SD)				
Serum calcium (mmol/L)	2.23 (0.185)	2.21 (0.20)	2.27 (0.16)	0.060^a^
Haemoglobin (g/dL)	10.6 (1.33)	10.7 (1.1)	10.6 (1.5)	0.781^a^
Serum albumin (g/L)	37 (5.5)	39 (4.5)	35 (5.6)	**< 0.001** ^a^
Dialysis adequacy (Kt/V)	–	1.66 (0.28) per dialysis	1.90 (0.37) per week	–
Wheelchair-bound				1.000^c^
Yes	5 (2.8)	3 (3.9)	2 (3.1)	
No	136 (97.2)	74 (96.1)	62 (96.9)	

*HD* Haemodialysis, *CAPD* Continuous ambulatory peritoneal dialysis, *CVD* cardiovascular disease, *SD* standard deviation, *SLE Systemic lupus erythematosus*

^a^Independent t-test; ^b^ Chi-Square test; ^c^ *Fisher’s exact test*

The mean age of the patients was 53.7 ± 14.20 years. Patients were predominantly male (55.3%), from Malay ethnicity (46.1%), with household income below RM3000 per month (80.9%), attained secondary education (59.6%) and unemployed (73.0%). The mean dialysis duration was 3.9 ± 1.43 years. Majority of the patients had hypertension (58.2%) and 48.9% had diabetes mellitus. Twenty patients (14.2%) had CVD (14.2%) and 51.8% were in normal BMI category. The mean serum calcium, haemoglobin and serum albumin was 2.243 ± 0.185 mmol/L, 10.6 ± 1.33 g/dL and 37 ± 5.5 g/L respectively. Dialysis adequacy (Kt/V) cannot be directly compared between HD and CAPD. HD patients had mean Kt/V of 1.66 ± 0.28 per dialysis and CAPD patients had mean Kt/V of 1.90 ± 0.37 per week. The groups differed significantly in terms of Hepatitis B and Hepatitis C status (*p* < 0.001) and mean serum albumin (*p* < 0.001). However, they did not differ in socio-demographic or other clinical characteristics.

### Utility index score and VAS score

The overall mean utility score for all patients was 0.877 ± 0.160 (0.905 ± 0.124 for CAPD and 0.854 ± 0.181 for HD, *p* = 0.157) and the mean VAS score was 76.6 ± 11.76 (77.1 ± 10.26 for CAPD and 76.2 ± 12.90 for HD, *p* = 0.921). Statistically significant differences were not found between HD and CAPD patients in both utility index score and VAS score (Table 2).

**Table 2:** *Utility index score and Visual* analogue score, haemodialysis and versus continuous ambulatory peritoneal dialysis

Score	All patients (*n* = 141)	HD (*n* = 77)	CAPD (*n* = 64)	*P* value
Utility index score				0.157^a^
mean (SD)	0.877 (0.160)	0.854 (0.181)	0.905 (0.124)	
Median (IQR)	0.880 (0.202)	0.880 (0.204)	1.000 (0.189)	
Minimum, Maximum	0.290,1.000	0.290,1.000	0.564,1.000	
VAS score				0.921^a^
mean (SD)	76.6 (11.76)	76.2 (12.90)	77.1 (10.26)	
Median (IQR)	80.0 (15.0)	80.0 (15.0)	80.0 (15.0)	
Minimum, Maximum	30,100	30,100	50,90	

^a^Mann Whitney U test*; IQR: Interquartile range*

### Correlation between utility score and VAS score

Assuming non-normal distribution of either one of the variables, a non-parametric test was used (Spearman Rank correlation) to determine the relationship between the predicted utility score and VAS score. There was a moderate, positive correlation between utility score and VAS score which was statistically significant (r = 0.677, *p* < 0.001).

### EQ-5D-3 L health domains

Table 3 shows the proportion of HD and CAPD patients reporting problems in each EQ-5D-3 L dimension. The highest proportion of problems was reported in the pain/discomfort dimension with 32.5% of HD patients and 35.9% of CAPD patients reporting problems. The lowest proportion of reported problems was observed in the self-care dimension, 11.3% of total patients. There was a significant difference of reported problems between HD and CAPD patients on usual activities dimension (*p* = 0.009**)** with 35.1 and 15.6% of patients reporting problems respectively.

**Table 3:** Proportions of problems reported on each EQ-5D dimension

Health Dimension	All patients (*n* = 141)	HD (*n* = 77)	CAPD (*n* = 64)	*P* value
Mobility, n (%)	37 (26.2)	23 (29.9)	14 (21.9)	> 0.05 ^a^
Self-Care, n (%)	16 (11.3)	11 (14.3)	5 (7.8)	> 0.05 ^a^
Usual Activities, n (%)	37 (26.2)	27 (35.1)	10 (15.6)	**0.009** ^a^
Pain / Discomfort, n (%)	48 (34.0)	25 (32.5)	23 (35.9)	> 0.05 ^a^
Anxiety / Depression, n (%)	20 (14.2)	11 (14.3)	9 (14.1)	> 0.05 ^a^

^a^*Chi*-*squared test (*χ 2)

### Bivariate analysis of independent factors and utility index score and VAS score

Table 4 shows the association of the independent factors with the EQ-5D-3 L utility score and VAS score. Factors significantly associated with HRQOL include presence of CVD, number of co-morbidities, haemoglobin, wheelchair-bound, age, diabetes mellitus and serum albumin.

**Table 4:** Bivariate analysis of independent factors and utility index score/visual analogue scale

Characteristics	Utility index score, mean (SD)	*p* value	VAS score, mean (SD)	*p* value
Modality		0.157^a^		0.921^a^
HD, *n* = 77	0.854		76.2	
CAPD, n = 64	0.905		77.1	
Age group, years		0.117^b^		**0.032** ^b^
Young, 18–45, *n* = 36	0.924		80.97	
Middle aged, 46–65 *n* = 75	0.850		74.66	
Elderly, > 65, *n* = 30	0.871		75.53	
Gender		0.764^a^		0.892^a^
Male, *n* = 78	0.871		76.06	
Female, *n* = 63	0.884		77.29	
Ethnicity		0.497^a^		0.787^a^
Malay, *n* = 65	0.873		76.23	
Non-Malay, *n* = 76	0.880		76.93	
Household income per month (RM)		0.453^a^		0.191^a^
≥ 3000, *n* = 27	0.908		79.26	
< 3000, *n* = 114	0.870		75.98	
Education level		0.376^b^		0.593^b^
Primary, *n* = 39	0.874		74.87	
Secondary, *n* = 84	0.871		77.64	
Tertiary, *n* = 18	0.911		75.56	
Occupation		0.261^a^		0.070^a^
Employed, *n* = 38	0.911		79.82	
Unemployed, *n* = 103	0.864		75.43	
Dialysis duration		0.508^a^		0.098^a^
≥ 4.0 years, =62	0.891		79.08	
< 4.0 years, *n* = 79	0.865		74.67	
Diabetes mellitus		0.191^a^		**0.035** ^a^
Diabetic, *n* = 69	0.857		74.09	
Non-diabetic, *n* = 72	0.896		79.03	
CVD		**0.001** ^a^		**0.028** ^a^
Present, *n* = 20	0.739		71.10	
Absent, *n* = 121	0.900		77.52	
Number of co-morbidities		**< 0.001** ^a^		**0.001** ^a^
< 3, *n* = 120	0.905		78.17	
≥ 3, *n* = 21	0.714		67.71	
Body mass Index (kg/m^2^)		0.834^a^		0.929^a^
< 25, *n* = 89	0.877		76.89	
≥ 25, *n* = 52	0.877		76.13	
Serum calcium (mmol/L)		0.340^a^		0.079^a^
< 2.20, *n* = 53	0.850		73.91	
≥ 2.20, *n* = 88	0.893		78.24	
Haemoglobin (g/dL)		**0.013** ^a^		0.164^a^
Low, < 10, *n* = 41	0.829		74.76	
High, ≥10, *n* = 100	0.896		77.37	
Serum albumin (g/L)		0.063^a^		**0.034** ^a^
Low, < 35, *n* = 50	0.836		73.10	
High, ≥35, *n* = 91	0.899		78.54	
Wheelchair-bound		**< 0.00** ^a^		**< 0.001** ^a^
Yes, n = 5	0.462		48.00	
No, *n* = 136	0.892		77.66	

^a^Mann Whitney U test ^b^ Kruskal-Wallis test

### Multivariate analysis of independent factors and utility index score

To adjust for the factors that were significantly associated with utility index score and VAS score in the bivariate analysis, one-way ANCOVA was used. The model for utility index score (Table 5) confirmed that the significant predictors of lower EQ-5D utility score included number of co-morbidities ≥3 (*p* < 0.004), low haemoglobin level < 10 g/dL (*p* = 0.002) and wheelchair-bound (*p* < 0.001). Wheelchair-bound status (*p* < 0.001) was a significant predictor of the lower VAS score (Table 6).

**Table 5:** Regression coefficients in generalised linear model of utility index score

Characteristics	Utility index score	
	*Adj. b* (95% CI)	*p* value
CVD		
Present	−0.070(− 0.142,0.002)	0.058
Absent (Ref)	–	–
Haemoglobin (g/dL)		
Low, < 10,	−0.073(− 0.118,-0.027)	**0.002**
High, ≥10 (Ref)	–	–
Wheelchair-bound		
Yes	−0.368(− 0.484,-0.253)	**< 0.001**
No (Ref)	–	–
Number of co-morbidities		
≥ 3	−0.106(,-0.179,-0.034)	**0.004**
< 3(Ref)	–	–

*Adj. b:* Standardised coefficients, CVD: Cardiovascular disease

Adjusted R^2^:39% (F 23.667; *p* < 0.001)

**Table 6:** Regression coefficients in generalised linear model of VAS score

Characteristics	VAS score	
	*Adj. b* (95% CI)	*p* value
Age group		
Middle aged, 46–65	− 3.419(− 7.283,0.986)	0.134
Elderly,> 65	− 5.295(− 10.602,0.012)	0.051
Young, 18–45 (Ref)	–	–
CVD		
Present	0.487(−5.531,6.506)	0.873
Absent (Ref)	–	–
Serum albumin (g/L)		
Low, < 35	−2.259(−6.211,1.513)	0.233
High, ≥35 (Ref)	–	–
Wheelchair-bound		
Yes	−25.567(−35.132,-16.003)	**< 0.001**
No (Ref)	–	–
Number of co-morbidities		
≥ 3	−5.963(−12.082, 0.155)	0.056
< 3(Ref)	–	–
Diabetes mellitus		
Diabetic	−0.715(−3.919,3.568)	0.926
Non-diabetic	–	–

*Adj. b:* Standardised coefficients, *CVD* Cardiovascular disease

Adjusted R^2^:27% (F 8.269; *p* < 0.001)

## Discussion

The present study investigates the HRQOL of HD and CAPD patients in 1 federal and 4 state hospitals in Malaysia. Statistically significant differences of utility index score and VAS score were not found between HD and CAPD patients. These findings are inconsistent with two earlier publications from Malaysia on dialysis related HRQOL. Liu et al. conducted a survey on 6908 HD and CAPD patients using Spitzer’s QOL index and HD was found to be a significant predictor for low HRQOL score [15]. In another study by Lui et al., a survey on 1332 dialysis patients was conducted at 15 dialysis centres using the World Health Organisation Quality of Life questionnaire (WHOQOL-BREF) and HD was identified as one of the significant predictors for low HRQOL score [16] The differences in study findings could be explained by several reasons. The large sample size in the previous studies probably enabled *small differences* to become statistically significant. In addition, the participation of a relatively large number of centres (*n* = 15) is perceived to introduce more variations in the results. The instruments used to measure the HRQOL were also different. However, the present study has provided the necessary utility index scores to calculate the QALY for a cost utility analysis to be carried out.

These results were consistent with other literature that used EQ-5D as their study instrument (or along with other study instruments). Among patients participating in a prospective cohort study on the adequacy of dialysis, de Wit et al. could not demonstrate differences between HD and PD patients using four different instruments including EQ-5D [25]. In Singapore, the closest country in terms of patients’ socio-demographic characteristics, dialysis modality has no impact on the health utility of HD and CAPD patients [17]. There are also other studies showing similar HRQOL between HD and CAPD patients using a variety of other instruments [26–33]. These findings were supported by a comprehensive systematic review by Liem et al. on preference based quality of life of patients on RRT. They did a meta-analysis of 27 articles including utilities from time-trade-off (TTO), standard gamble (SG), and EQ-5D studies and found that there were no statistically significant differences in HRQOL between HD and PD patients [34]. The insignificant differences in HRQOL suggests that the relative cost-effectiveness of the two dialysis modalities in Malaysia would be mainly determined by their associated costs.

A significant difference of problems reported in the usual activity domain was identified between the two modalities in this study where CAPD patients reported less problems as compared to HD patients. This is not surprising since PD patients scored higher in physical activity domain in previous literature [35–39]. CAPD is often perceived as the easier and less burdensome dialysis modality since dialysis may be performed at home. In contrast, HD patients have to travel to their respective dialysis centres to get treatment and would usually remain there for four hours, thus restricting their daily activities. However findings are mixed; some researchers showed that patients on HD scored better in physical activity domain as compared to patients on PD [28, 33, 40, 41]. In fact, HD patients tend to improve in their daily activities as they continue the treatment [33].

The high proportion of problems reported in pain/discomfort domain is of concern. It was observed in patients on both modalities. There are many factors that can lead to either acute or chronic pain in patients on HD, as well as in patients on CAPD. Pain/discomfort have been shown to influence the overall quality of life HD and CAPD patients [32, 42]. Theoretically this could be attributed to the pain due to dialysis access and needles, as well as existing co-morbidities. It would be hypothetical to come to any kind of conclusion about the reasons for the high proportion of pain/discomfort found in this group of patients. This result indicates that a separate study with the focus on body pain should be conducted to address the limited studies found in literature.

In the one-way ANCOVA, low haemoglobin level (< 10 g/dL), number of co-morbidities ≥3 and wheelchair-bound status were significant predictors for poor HRQOL. Similar associations were observed in previous studies [15–17, 43–47]. Lopes et al. found that co-morbidity, and low haemoglobin level were among the factors independently and significantly associated with impaired physical health in 9000 HD patients from seven countries [43]. Finkelstein et al. (2009) asserted that the energy/vitality domain, the physical composite score of the SF-36, and the general health score of 1200 patients with stage 3, 4, and 5 Chronic kidney disease (CKD) increased significantly with hemoglobin levels elevation [44]. A study of PD patients using EQ-5D instrument showed that significant predictors of high VAS score included reduced co-morbidities and use of erythropoietin [45]. Recently, a cross sectional survey by Eriksson et al. among CKD patients across Europe emphasised that after stratification by anaemia status, impairment was consistently lower for anaemic than non-anaemic CKD patients across various measurement scales [46].

The impact of CVD in dialysis patients is well established. CVD is a predictor of poor HRQOL in patients with ESRD [47]. Registry studies confirmed that CVD is an independent risk factor for and the leading cause of death in dialysis patients, accounting for nearly 50% of deaths in this population [7, 45–49]. However, CVD alone was not found to be a significant predictor of HRQOL in this research possibly due to the presence of additional co-morbidities. Wheelchair-bound patients have obvious restrictions in morbidity and physical functioning. They were more likely to report poor health than people without a disability [50, 51].

Remarkably, the mean utility score for patients in this study was high (overall = 0.877, HD = 0.854, CAPD = 0.905) compared to other literature using EQ-5D as their main instrument. Typical values of prevalent dialysis patients are in the 0.40–0.70 range [14, 17, 25, 34]. These observations could be attributed to several factors. First and foremost, a Malaysian value set was used to calculate utility index scores. There are different value sets developed in different countries and each value set carry different weights. For example, 11,112 (mobility = 1, self-care = 1, usual activities = 1, pain/discomfort = 1, and anxiety/depression = 2), yields 0.852, 0.782 and 0.799 using Malaysian value set, UK value set and Spanish value set respectively (VAS as referent). The methodological dissimilarities among other cultural divergence in the valuation studies across countries conceivably prompted the difference in the utility index scores [52]. The higher utility scores from the Malaysian value set are also related to the valuation study where patient valuations was used instead of the general population [23]. Valuation studies in Singapore, Thailand, and the UK used the general population as respondents [53]. A similar observation was reported in Sweden when higher utility scores were derived from the study using patient valuations instead of the general population [54]. Zhao et al. supported that country-specific tariff should be used since there were significant differences among the three national tariff sets (Chinese, UK, Japanese) [55]. Another possible reason is that, half of the respondents in this study reported no problems in all health states (11111). Patients in this study were perceived to receive better care since they were recruited from state hospitals. The decision to use the Malaysian value set is justified. In a study by Endarti et al. that compared EQ-5D-3 L index scores using Malaysian, Singaporean, Thai, and the UK value sets in Indonesian cervical cancer patients, the Malaysian value set achieved the highest interrelation intraclass correlation coefficients (ICC) versus VAS scores compared with the ICCs of the other three scores versus VAS scores [52].

This study has several limitations. First, a causal relationship could not be established between certain variables and HRQOL since it was a cross-sectional study. Second, this study was conducted in only five large hospitals which could have had a positive influence on the overall HRQOL. The experiences of these patients may differ with those of patients managed in other dialysis centres e.g. in district hospitals. Future HRQOL study among dialysis patients using EQ-5D questionnaire in Malaysia should include more centres to introduce variability in the sample as shown in previous studies. Besides, the sample size is relatively small as compared to other studies. Lastly. there are other factors that were not studied including cultural, religious practices and environment which could affect HRQOL as indicated in the low adjusted R^2^.

## Conclusions

In conclusion, the present study provides an understanding of overall HRQOL of HD and CAPD patients using the EQ-5D questionnaire. The results show that dialysis modality had no impact on HRQOL although PD patients scored a higher utility index. Other factors including low haemoglobin level, number of co-morbidities and wheelchair-bound status were significant predictors of low HRQOL. Health domains profoundly affected include usual activities and pain/discomfort domains. This study indicates that the EQ-5D questionnaire can both measure patients’ HRQOL in local settings and produce QALYs as key outcome measure in economic evaluation studies.

## Abbreviations

BMI: Body mass index
CAPD: Continuous ambulatory peritoneal dialysis
CKD: Chronic kidney disease
CVD: Cardiovascular disease
DALY: Disability adjusted life year
ESRD: End stage renal disease
GFR: Granular filtration rate
HD: Haemodialysis
HRQOL: Health related quality of life
MOH: Ministry of Health
PD: Peritoneal dialysis
QALY: Quality adjusted life year
QOL: Quality of life
RRT: Renal replacement therapy
SD: Standard deviation
SG: Standard gamble
SLE: *Systemic lupus erythematosus*
TTO: Time trade off
VAS: Visual analogue scale
VIF: Variance inflation factors
WHO: World Health Organisation
WHOQOL-BREF: World Health Organisation Quality of Life questionnaire

## References

[1] Jaar BG, Chang A, Plantinga L (2013). Can we improve quality of life of patients on dialysis?. Clin J Am Soc Nephrol.

[2] Megari K (2013). Quality of life in chronic disease patients. Health Psychol Res.

[3] Wang HM, Beyer M, Gensichen J, Gerlach FM (2008). Health-related quality of life among general practice patients with differing chronic diseases in Germany: Cross sectional survey. BMC Public Health.

[4] EBPG (European Expert Group on Renal Transplantation) (2000). European Renal Association (ERA-EDTA), European Society for Organ Transplantation (ESOT). European best practice guidelines for renal transplantation (Part 1). Nephrol Dial Transplant.

[5] Sennfalt K, Magnusson M, Carlsson P (2002). Comparison of hemodialysis and peritoneal dialysis—a cost-utility analysis. Perit Dial Int.

[6] Abdul Manaf MR, Surendra NK, Abdul Gafor AH, Seong Hooi L, Bavanandan S (2017). Dialysis Provision and Implications of Health Economics on Peritoneal Dialysis Utilization: A Review from a Malaysian Perspective. Int J Nephrol.

[7] Wong HS, Goh BL (2017). 23^rd^ Report of the Malaysian Dialysis and Transplant Registry.

[8] Thomas R, Kanso A, Sedo J (2008). Chronic kidney disease and its complications. Prim Care.

[9] World Health Organization (2015). Global Health Estimates 2000-2015.

[10] Joshi VD (2014). Quality of life in end stage renal disease patients. World J Nephro.

[11] Malindretos P (2012). Health Related Quality of Life in Chronic Kidney Disease Patients. J Palliat Care Med.

[12] Unruh ML, Hess R (2007). Assessment of health-related quality of life among patients with chronic kidney disease. Adv Chronic Kidney Dis.

[13] Valderrábano F, Jofre R, López JM (2001). Quality of life in end-stage renal disease patients. Am J Kidney Dis.

[14] Wyld M, Morton R, Hayen A, Howard K, Webster AC (2012). A Systematic Review and Meta-Analysis of Utility-Based Quality of Life in Chronic Kidney Disease Treatments. PLoS Med.

[15] Liu WJ, Chew TF, Chiu AS, Zaki M (2006). Quality of life of dialysis patients in Malaysia. Med J Malaysia.

[16] Liu WJ, Musa R, Chew TF, Lim CTS, Morad Z, Bujang A (2014). Quality of life in dialysis: A Malaysian perspective. Hemodial Int.

[17] Yang F, Griva K, Lau T (2015). Health-related quality of life of Asian patients with end-stage renal disease (ESRD) in Singapore. Qual Life Res.

[18] Gentile S, Delarozière JC, Fernandez C, Tardieu S, Devictor B, Dussol B (2003). Review of quality of life instruments used in end-stage renal disease. Nephrologie.

[19] Md Yusop NB, Yoke Mun C, Shariff ZM, Beng Huat C (2013). Factors Associated with Quality of Life among Hemodialysis Patients in Malaysia. PLoS One.

[20] Noyes J, Edward RT (2011). EQ-5D for the Assessment of Health-Related Quality of Life and Resource Allocation in Children: A Systematic Methodological Review. Value Health.

[21] Euroqol. EQ-5D. https://euroqol.org/. Accessed 30 Jan 2018.

[22] Yusof FAM, Goh A, Azmi S (2012). Estimating an EQ-5D Value Set for Malaysia Using Time Trade-Off and Visual Analogue Scale Methods. Value Health.

[23] National Institute for Health and Clinical Excellence (NICE) (2008). Guide to the methods of technology appraisal. London: NICE.

[24] Hochberg MC (1997). Updating the American College of Rheumatology revised criteria for the classification of systemic lupus erythematosus. Arthritis Rheum.

[25] de Wit GA, Merkus MP, Krediet RT, de Charro FT (2002). Health profiles and health preferences of dialysis patients. Nephrol Dial Transplant.

[26] Bass EB, Wills S, Fink NE (2004). How strong are patients' preferences in choices between dialysis modalities and doses?. Am J Kidney Dis.

[27] De Abreu MM, Walker DR, Sesso RC, Ferraz MB (2011). Health-Related Quality of Life of Patients Recieving Hemodialysis and Peritoneal Dialysis in São Paulo, Brazil: A Longitudinal Study. Value Health.

[28] Griva K, Kang AW, Yu ZL (2014). Quality of life and emotional distress between patients on peritoneal dialysis versus community-based hemodialysis. Qual Life Res.

[29] Harris SA, Lamping DL, Brown EA, Constantinovici N (2002). Clinical outcomes and quality of life in elderly patients on peritoneal dialysis versus hemodialysis. Perit Dial Int.

[30] Manns B, Johnson JA, Taub K, Mortis G, Ghali WA, Donaldson C (2003). Quality of life in patients treated with hemodialysis or peritoneal dialysis: what are the important determinants?. Clin Nephrol.

[31] Peng YS, Chiang CK, Hung KY (2011). Comparison of self-reported health-related quality of life between Taiwan hemodialysis and peritoneal dialysis patients: a multi-center collaborative study. Qual Life Res.

[32] Wasserfallen JB, Halabi G, Saudan P (2004). Quality of life on chronic dialysis: comparison between hemodialysis and peritoneal dialysis. Nephrol Dial Transplant.

[33] Wu AW, Fink NE, Marsh-Manzi JV (2004). Changes in Quality of Life during Hemodialysis and Peritoneal Dialysis Treatment: Generic and Disease Specific Measures. J Am Soc Nephrol.

[34] Liem YS, Bosch JL, Hunink M (2008). Preference-Based Quality of Life of Patients on Renal Replacement Therapy: A Systematic Review and Meta-Analysis. Value Health.

[35] Atapour A, Nasr S, Boroujeni AM, Taheri D, Dolatkhah S (2016). A comparison of the quality of life of the patients undergoing hemodialysis versus peritoneal dialysis and its correlation to the quality of dialysis. Saudi J Kidney Dis Transpl.

[36] Ginieri-Coccossis M, Theofilou P, Synodinou C, Tomaras V, Soldatos C (2008). Quality of life, mental health and health beliefs in hemodialysis and peritoneal dialysis patients: Investigating differences in early and later years of current treatment. BMC Nephrol.

[37] Kutner NG, Zhang R, Barnhart H, Collins AJ (2005). Health status and quality of life reported by incident patients after 1 year on hemodialysis or peritoneal dialysis. Nephrol Dial Transplant.

[38] Makkar V, Kumar M, Mahajan R, Khaira NS (2015). The Comparison of Quality of Life among Peritoneal and Hemodialysis Patients. J Clin Diagn Res.

[39] Theofilou P (2011). Quality of Life in Patients Undergoing Hemodialysis or Peritoneal Dialysis Treatment. J Clin Med Res.

[40] Al Wakeel J, Al Harbi A, Bayoumi M, Al-Suwaida K, Al Ghonaim M, Mishkiry A (2012). Quality of life in hemodialysis and peritoneal dialysis patients in Saudi Arabia. Ann Saudi Med.

[41] Diaz-Buxo JA, Lowrie EG, Lew NL, Zhang H, Lazarus JM (2000). Quality-of-life evaluation using Short Form 36: comparison in hemodialysis and peritoneal dialysis patients. Am J Kidney Dis.

[42] Aghakhani N, Sharif Nia H, Samad Zadeh S, Toupchi V, Toupchi S, Rahbar N (2011). Quality of life during hemodialysis and study dialysis treatment in patients referred to teaching hospitals in Urmia-Iran in 2007. Caspian J Intern Med.

[43] Lopes AA, Bragg-Gresham JL, Goodkin DA (2007). Factors associated with health-related quality of life among hemodialysis patients in the DOPPS Qual. Life Res.

[44] Finkelstein FO, Story K, Firanek C (2009). Health-Related Quality of Life and Hemoglobin Levels in Chronic Kidney Disease Patients. Clin J Am Soc Nephrol.

[45] Sakthong P, Kasemsup V (2012). Health utility measured with EQ-5D in Thai patients undergoing peritoneal dialysis. Value Health.

[46] Eriksson D, Goldsmith D, Teitsson S, Jackson J, van Nooten F (2016). Cross-sectional survey in CKD patients across Europe describing the association between quality of life and anaemia. BMC Nephrol.

[47] Pagels AA, Söderkvist BK, Medin C, Hylander B, Heiwe S (2012). Health-related quality of life in different stages of chronic kidney disease and at initiation of dialysis treatment. Health Qual Life Outcomes.

[48] Locatelli F, Pozzoni P, Tentori F, Del VL (2003). Epidemiology of cardiovascular risk in patients with chronic kidney disease. Nephrol Dial Transplant.

[49] United States Renal Data Systems (USRDS) (2018). 2017 Annual Data Report.

[50] Forman-Hoffman VL, Ault KL, Anderson WL, Weiner JM, Stevens A, Campbell VA, Armour BS (2015). Disability Status, Mortality, and Leading Causes of Death in the United States Community Population. Med Care.

[51] Krahn GL, Walker DK, Correa-De-Araujo R (2015). Persons with Disabilities as an Unrecognized Health Disparity Population. Am J Public Health.

[52] Knies S, Evers SM, Candel MJ, Severens JL, Ament AJ (2009). Utilities of the EQ-5D: Transferable or not. Pharmacoeconomics.

[53] Endarti D, Riewpaiboon A, Thavorncharoensap M, Praditsitthikorn N, Hutubessy R, Kristina SA (2018). A Comparison of EQ-5D-3L Index Scores Using Malaysian, Singaporean, Thai, and UK Value Sets in Indonesian Cervical Cancer Patients. Value Health Reg Issues.

[54] Kiadaliri A, Eliasson B, Gerdtham UG (2015). Does the choice of EQ-5D tariff matter? A comparison of the Swedish EQ-5D-3L index score with UK, US, Germany and Denmark among type 2 diabetes patients. Health Qual Life Outcomes.

[55] Zhao Y, Li S-P, Liu L, Zhang J-L, Chen G (2017). Does the choice of tariff matter? A comparison of EQ-5D-5L utility scores using Chinese, UK, and Japanese tariffs on patients with psoriasis vulgaris in Central South China. Medicine (Baltimore).

